# Targeted Disruption of *Chlamydia trachomatis* Invasion by in Trans Expression of Dominant Negative Tarp Effectors

**DOI:** 10.3389/fcimb.2016.00084

**Published:** 2016-08-23

**Authors:** Christopher J. Parrett, Robert V. Lenoci, Brenda Nguyen, Lauren Russell, Travis J. Jewett

**Affiliations:** Division of Immunology and Pathogenesis, Burnett School of Biomedical Sciences, College of Medicine, University of Central FloridaOrlando, FL, USA

**Keywords:** chlamydia, invasion, cytoskeleton, type III secretion, effectors, Tarp

## Abstract

*Chlamydia trachomatis* invasion of eukaryotic host cells is facilitated, in part, by the type III secreted effector protein, Tarp. The role of Tarp in chlamydiae entry of host cells is supported by molecular approaches that examined recombinant Tarp or Tarp effectors expressed within heterologous systems. A major limitation in the ability to study the contribution of Tarp to chlamydial invasion of host cells was the prior absence of genetic tools for chlamydiae. Based on our knowledge of Tarp domain structure and function along with the introduction of genetic approaches in *C. trachomatis*, we hypothesized that Tarp function could be disrupted *in vivo* by the introduction of dominant negative mutant alleles. We provide evidence that transformed *C. trachomatis* produced epitope tagged Tarp, which was secreted into the host cell during invasion. We examined the effects of domain specific Tarp mutations on chlamydial invasion and growth and demonstrate that *C. trachomatis* clones harboring engineered Tarp mutants lacking either the actin binding domain or the phosphorylation domain had reduced levels of invasion into host cells. These data provide the first *in vivo* evidence for the critical role of Tarp in *C. trachomatis* pathogenesis and indicate that chlamydial invasion of host cells can be attenuated via the introduction of engineered dominant negative type three effectors.

## Introduction

*Chlamydia trachomatis* is an obligate intracellular bacterium responsible for many human diseases (Moulder et al., 1984). Distinct serovars are the etiologic agents of endemic blinding trachoma, sexually transmitted disease, and lymphogranuloma venereum (Byrne, 2010). Chlamydiae undergo a unique developmental cycle consisting of two metabolically and morphologically distinct developmental forms adapted for extracellular survival and intracellular multiplication, respectively (Swanson et al., 1975; Szaszak et al., 2011; Omsland et al., 2012). Elementary bodies (EBs) are small, metabolically dormant cell types that actively promote invasion of eukaryotic host cells (Carabeo et al., 2002). Reticulate bodies (RBs) are larger cell types that are metabolically active and undergo replication (Omsland et al., 2012). EBs differentiate into RBs within the first few hours following infection. The RBs then multiply by binary fission until ~16–24 h post-infection, at which time they asynchronously begin to differentiate back into EBs prior to release from the host cell and initiation of subsequent rounds of infection (Moulder et al., 1984).

Like many Gram-negative pathogens, chlamydiae have a type III secretion system (T3SS) which they utilize to translocate various effector proteins into the cytosol of the host cell. Additionally, some secreted effectors localize to the expanding inclusion membrane and are collectively referred to as the Inc., proteins (Coburn et al., 2007). The chlamydial T3SS functions in at least two distinct locations and times during chlamydial development (Muschiol et al., 2006; Betts-Hampikian and Fields, 2010; Case et al., 2010). One pool of early effectors, pre-existing in EBs, is secreted upon contact with a host cell without a requirement for chlamydial protein synthesis (Jamison and Hackstadt, 2008; Valdivia, 2008). Later in the developmental cycle, other effectors are secreted out toward the cytosol from within the inclusion after initiation of protein synthesis (Wolf et al., 2006). The translocated actin-recruiting phosphoprotein (Tarp) is one of the early effectors and is spatially and temporally associated with the recruitment of actin to the site of EB invasion (Clifton et al., 2004). Tarp is phosphorylated upon translocation into eukaryotic cells by host tyrosine kinases (Jewett et al., 2008; Mehlitz et al., 2008). All isolates of pathogenic *Chlamydia* examined to date harbor the *tarP* gene (Clifton et al., 2005; Lutter et al., 2010). Biochemical analysis of *C. trachomatis* Tarp and other Tarp orthologs revealed that Tarp is comprised of an actin nucleating domain which is conserved and a tyrosine-rich repeat domain that is specific to serovars of *C. trachomatis* (Clifton et al., 2005; Jewett et al., 2006, 2010). Tarp associates directly with both globular (G-) and filamentous (F-) actin via small alpha helical domains contained within the C-terminal region of the protein (Jewett et al., 2006, 2010; Jiwani et al., 2013). Tarp's ability to directly bind to actin contributes to two biochemically characterized functions, actin nucleation and actin bundling, which likely lead to cytoskeletal modifications in the target host cell during entry (Jewett et al., 2006; Jiwani et al., 2013). Tarp independently nucleates new actin filaments by forming a large homogenous multimeric protein complex mediated by a conserved proline rich domain (Jewett et al., 2006). Inhibition of the actin binding alpha helix with microinjected antibodies specific for the Tarp actin binding domain blocked Tarp-mediated actin polymerization *in vitro* and reduced *C. trachomatis* L2 entry into host cells, suggesting Tarp is a critical virulence factor associated with chlamydial invasion (Jewett et al., 2010).

Although the direct actin-nucleating potential of *C. trachomatis* Tarp is implicated in bacterial entry of host cells, other actin nucleating pathways involving activation of the Arp2/3 complex are also necessary for entry (Carabeo et al., 2007; Jewett et al., 2010). Interestingly, phosphorylated Tarp may also play a role by indirectly activating the Arp2/3 complex as Tarp immunoprecipitation and peptide array assays have identified host cell signaling proteins such as Eps8, Rac1, Abi1, Sos1, Vav2, and SHC1 that associate with phosphorylated Tarp and promote Arp2/3 activation (Lane et al., 2008; Mehlitz et al., 2010). Biochemically, the actin nucleating properties of Tarp and the Arp2/3 complex work together to rapidly form actin filaments required for internalization (Jiwani et al., 2012). However, the precise details of how these distinct pathways cooperate to promote chlamydial internalization still remains unclear.

Since the Tarp effector has been characterized by various cellular and molecular approaches, and is implicated in chlamydial invasion of host cells, we sought to engineer mutant Tarp effectors that would biochemically interfere with endogenous Tarp function *in vivo*. In this work we examined *C. trachomatis* transformants expressing epitope tagged mutant Tarp alleles for their ability to invade host cells. Here, we report that EBs which secrete mutant Tarp effectors harboring specific domain deletions are deficient in bacterial entry of host cells. As hypothesized, those EBs which expressed a mutant Tarp lacking the actin binding domain, required for actin nucleation, were attenuated for bacterial invasion of host cells. Interestingly, the greatest inhibition of chlamydial entry was observed for those EBs which expressed Tarp effectors lacking the phosphorylation domain. These findings strongly support a role for Tarp in pathogen entry of host cells, and suggests that expression and delivery of engineered dominant negative mutant effectors may be employed to attenuate *C. trachomatis* pathogenesis.

## Materials and methods

### Organisms and cell culture

*C. trachomatis* serovar L2 (LGV 434) was propagated in HeLa 229 cells (ATCC CCL-2.1) or McCoy B cells (ATCC CRL-1696) and purified by Renografin density gradient centrifugation (Scidmore, 2005).

### Cloning and *C. trachomatis* transformations

In previous studies we had generated a number of in-frame Tarp deletions which were expressed as mutant GST-Tarp fusion proteins from pGEX-6p-1 (GE Health Sciences) plasmids (Jiwani et al., 2013). Tarp domain deletion mutants included: phosphorylation domain deletion (Δphos; deletion of D125 to Y424), proline rich domain deletion (ΔPRD; deletion of S625 to N650), actin binding domain deletion (ΔABD; A748 to K758), and F-actin binding domain 1 and 2 deletion (ΔFAB 1&2; deletion of L871 to G1005). These mutant Tarp alleles were subcloned into the chlamydial shuttle vector pCtSV.1 in a two-step process. First, wild type Tarp sequence was amplified from *C. trachomatis* (LGV 434) genomic DNA (Qiagen genomic purification kit, Valencia, CA). The forward (5′ACTCCGCGGTATTGCAT TTCTTCACAAACGTTACC-3′) and reverse (5′TATA TACAATTGTTACAGGTCCTCTTCAGATATTAGTTTTTGTTCTCCTACGGTATCAATCAGTG AGC-3′) DNA primers (Integrated DNA Technologies, Coralville, IA) were engineered to amplify 200 bases of putative Tarp promoter sequence and an in frame 3′ c-myc epitope tag by PCR with SacII and MfeI linkers. PCR products were purified (Qiagen), digested with restriction enzymes (New England Biolabs, Beverly, MA) and cloned into linearized pCtSV.1. This procedure resulted in the parent pCtSV.Tarp plasmid in which all other plasmids engineered to express Tarp mutants were generated. pCtSV.Tarp mutant derivatives were generated by exchanging the mutant DNA sequence from those pGEX-6p-1 clones described above. For example, pCtSV.TarpΔphos resulted from DNA exchange with digested Tarp DNA sequence flanking the phosphorylation domain with restriction sites BstAP1 and BmgB1 from pGEX-6p-1 TarpΔphos. Similarly, the other pCtSV.Tarp mutant clones were generated albeit with unique restriction enzymes which flanked the corresponding domain: The proline rich domain with BmgB1 and Bsm1, the actin binding domain with Bsm1 and Nco1, and the F-actin binding domains 1 and 2 with Nco1 and Mfe1. All engineered vectors were confirmed to be free of extraneous mutations by DNA sequence analysis and all in frame domain deletions were verified. All chlamydial shuttle vectors were purified from *E. coli* K12 ER2925 cells (New England Biolabs) and transformed into *C. trachomatis* (LGV 434) (Wang et al., 2011). All *C. trachomatis* transformants were density gradient purified and the number of infectious (inclusion) forming units (IFUs) was determined by indirect immunofluorescence of infected host cells with serially diluted purified elementary bodies (EBs).

### Pyrene assay

Pyrene actin polymerization assays were performed as previously described (Jiwani et al., 2013). Briefly, monomeric pyrene-labeled actin was prepared by diluting 100 μg of lyophilized pyrene actin (cytoskeleton Inc. Denver, CO) in 2 mL of 5 mM Tris (pH 8.0), 0.2 mM CaCl_2_, 0.2 mM ATP (G buffer) and incubated for 1 h at room temperature, followed by an additional 1 h incubation at 4°C. Monomeric pyrene actin was obtained by collecting the supernatant after a 2 h 100,000 × g 4°C spin in a Beckman Optima TLX Ultracentrifuge using a TLA 100.3 rotor (Beckman Coulter). Approximately 20 μg of pyrene-labeled actin was gently mixed with 5 μg of GST fusion proteins in a volume of 500 μL for 10 min before the addition of 1/20th volume of polymerization buffer (500 mM KCl, 20 mM MgCl_2_, 10 mM ATP). The reaction was monitored over 1 h with an LS 55 Luminescence spectrophotometer directed by FL WinLab software version 4.0 (Perkin-Elmer, Beaconsfield, Bucks, United Kingdom) with 2.5-nm bandwidth at 365-nm excitation wavelength and 2.5-nm bandwidth at 407-nm emission wavelength.

### SDS-PAGE and immunoblotting

Proteins were separated on SDS 5–15% polyacrylamide gels (BIORAD, Hercules, CA) and either stained with Imperial protein stain (Pierce, Rockford, IL) or transferred to 0.45 μm pure nitrocellulose transfer and immobilization membrane (Schleicher and Schuell, Keene, NH). Immunoblotting employed peroxidase conjugated secondary antibodies (Chemicon International, Temecula, CA) and Supersignal West Pico chemiluminescent substrate (Pierce). The anti-actin C4 monoclonal antibody was purchased from Chemicon International. The anti-actin polyclonal antibody was purchased from Cytoskeleton, Inc. The anti-phosphotyrosine 4G10 monoclonal antibody was purchased from Upstate (Millipore). The anti-chlamydial EB polyclonal antibody, the Momp monoclonal antibody and the GAPDH monoclonal antibody were all purchased from Pierce. The anti-c-myc monoclonal antibody was purchased from Genscript (Piscataway, NJ). The anti-chlamydial Hsp60 A57-B9 monoclonal antibody was purchased from Thermo Fisher Scientific (Waltham, MA). Polyclonal rabbit antibodies directed toward *C. trachomatis* L2 LGV 434 Tarp (CT456) were developed at Rocky Mountain Laboratories as previously described (Clifton et al., 2004).

### Invasion assay and indirect immunofluorescence microscopy

Intrinsically fluorescent EBs from *C. trachomatis* transformants were purified from cell cultures supplemented with CellTracker™ Red CMTPX Dye as previously described (Carabeo et al., 2007). Briefly, CMPTX-labeled *C. trachomatis* EBs (MOI ~50) were permitted to attach to HeLa 229 host cells for 30 min at 4°C. HeLa 229 cells were prepared in 24 well plates with cover slips and grown in Dulbecco's modified Eagle's medium (DMEM) containing 10% fetal bovine serum (FBS) and 1% L-glutamine for 24 h prior to infection. The cultures were rinsed with cold HBSS and the temperature shifted to 37°C by the addition of pre-warmed DMEM plus 10% FBS. The cultures were then incubated at 37°C for 1 h. The cultures were fixed with 4% paraformaldehyde at room temperature for 15 min and rinsed with PBS. The cells were not permeabilized. Extracellular EBs were labeled for 1 h with a monoclonal antibody specific for chlamydial major outer membrane protein (MOMP). After four washes in PBS, secondary antibody conjugated to Alexa 488 was added for 1 h. Coverslips were rinsed and mounted in ProLong Gold antifade reagent (Invitrogen, Carlsbad, CA). Cells were examined with a Zeiss Axio Observer A1 microscope equipped with a phase-contrast and epifluorescence optics. Images were obtained using an AxioCam MRm camera controlled by Axio Vision 4.8.2 and further processed using Adobe Photoshop CS2. The number of green (external) and red (total) EBs was determined for each host cell. These data were then used to determine the percentage of internalized EBs. Twenty fields of view were taken from each cover slip and these percentages were then averaged together to give a final invasion rate.

### Subcellular fractionation and protein extraction

*C. trachomatis* infected cells underwent subcellular fractionation as previously described (Cox and Emili, 2006). Briefly, *C. trachomatis* infected McCoy or HeLa 229 cells maintained at 37 or 4°C or host cells alone incubated at 37°C were removed from flasks and suspended in 100 mM KCl, 10 mM HEPES (pH 7.7), 2 mM MgCl_2_, and 2 mM ATP (Buffer A) and disrupted by sonication delivered in three consecutive 30 s intervals (~ 2000 joules) using an ultrasonic sonicator processor XL equipped with a microtip (Misonix Incorporated, Farmingdale, NY). All cell lysates underwent subcellular fractionation by sequential centrifugation in which supernatants and pellets were separated. Lysates were initially subject to an 800 × g spin for 15 min at 4°C. The 800 × g supernatants were then subjected to a 10,000 × g spin for 30 min at 4°C. The remaining 10,000 × g supernatant underwent a 100,000 × g spin for 1 h at 4°C. Protein sample buffer was added to all pellets and supernatants and proteins were resolved by SDS-PAGE and transferred to nitrocellulose membranes for immunoblotting with antibodies specific for c-myc, Tarp, actin, GAPDH, Momp, and chlamydial EBs. Subcellular fractionation experiments were conducted in both McCoy (**Figure 6**) and HeLa 229 (Supplemental Figure 1) cells.

### *C. trachomatis* development

HeLa 229 cells were seeded into 6 well plates (2 × 10^5^ cells/well) and grown in Dulbecco's modified Eagle's medium (DMEM) containing 10% fetal bovine serum (FBS) and 1% L-glutamine for 24 h. Individual wells were infected with wild type *C. trachomatis* L2 (LGV 434) or *C. trachomatis* transformants. All host cells and bacteria were collected from select wells (cells scraped off the bottom of each well and collected in 15 mL tubes, and sonicated for 30 s using a microtip equipped Misonix sonicator) at 0, 12, 24, 36, and 48 h. Cell lysates were then frozen at −80°C until all time points had been collected. Cell lysates were thawed on ice and diluted and then placed onto HeLa cells grown on 16 mm circular cover slips contained within 24 well plates and grown in Dulbecco's modified Eagle's medium (DMEM) supplemented with 10% fetal bovine serum (FBS) and 1% L-glutamine. After a 40 h incubation, infected cells were then immunostained and observed under a fluorescent microscope for inclusion formation. Twenty fields of view were taken from each cover slip (the experiment was performed in triplicate) and cover slip counts were averaged. Averages were plotted using GraphPad Prism software.

## Results

### Mutant tarp proteins diminish the ability of wild type tarp to polymerize actin filaments

**Figure 1:**
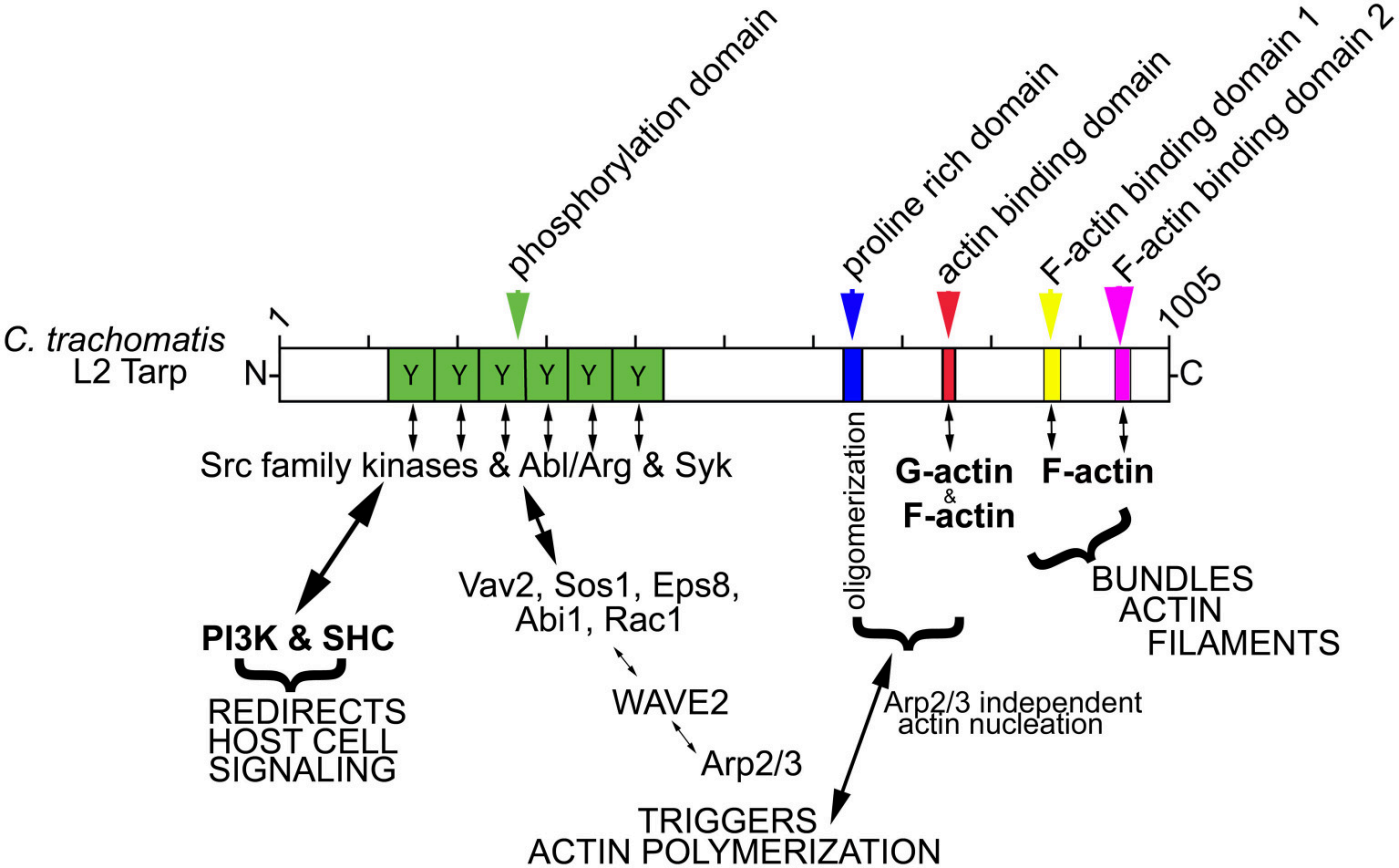
**Schematic of Tarp protein mediated signaling pathways implicated in chlamydial entry and development**. *C. trachomatis* Tarp harbors an N-terminal tyrosine rich repeat phosphorylation domain (Y, green box). Tyrosine residues are phosphorylated by members of the Src family kinases (SFKs) such as Src, Yes and Fyn and by other tyrosine kinases, Syk and Abl/Arg kinases. The actin nucleating activity of Tarp results from distinct G-actin binding (red box) and proline rich (blue box) oligomerization domains and from an Arp2/3-dependent pathway resulting from the recruitment and activation of host cell signaling proteins Vav2, Sos1, Eps8, Abi1, Rac1, and WAVE2. Tarp also harbors two F-actin binding domains (FAB 1 and 2: yellow and pink boxes) that are implicated in the formation of actin bundles. Phosphorylated Tarp can also associate with the host cell Src homology 2 domain containing protein 1 (SHC1) and the phosphoinositide 3-kinase (PI3K), which are implicated in altering the activation state of host signaling proteins to create a protective niche for the developing bacterium.

**Figure 2:**
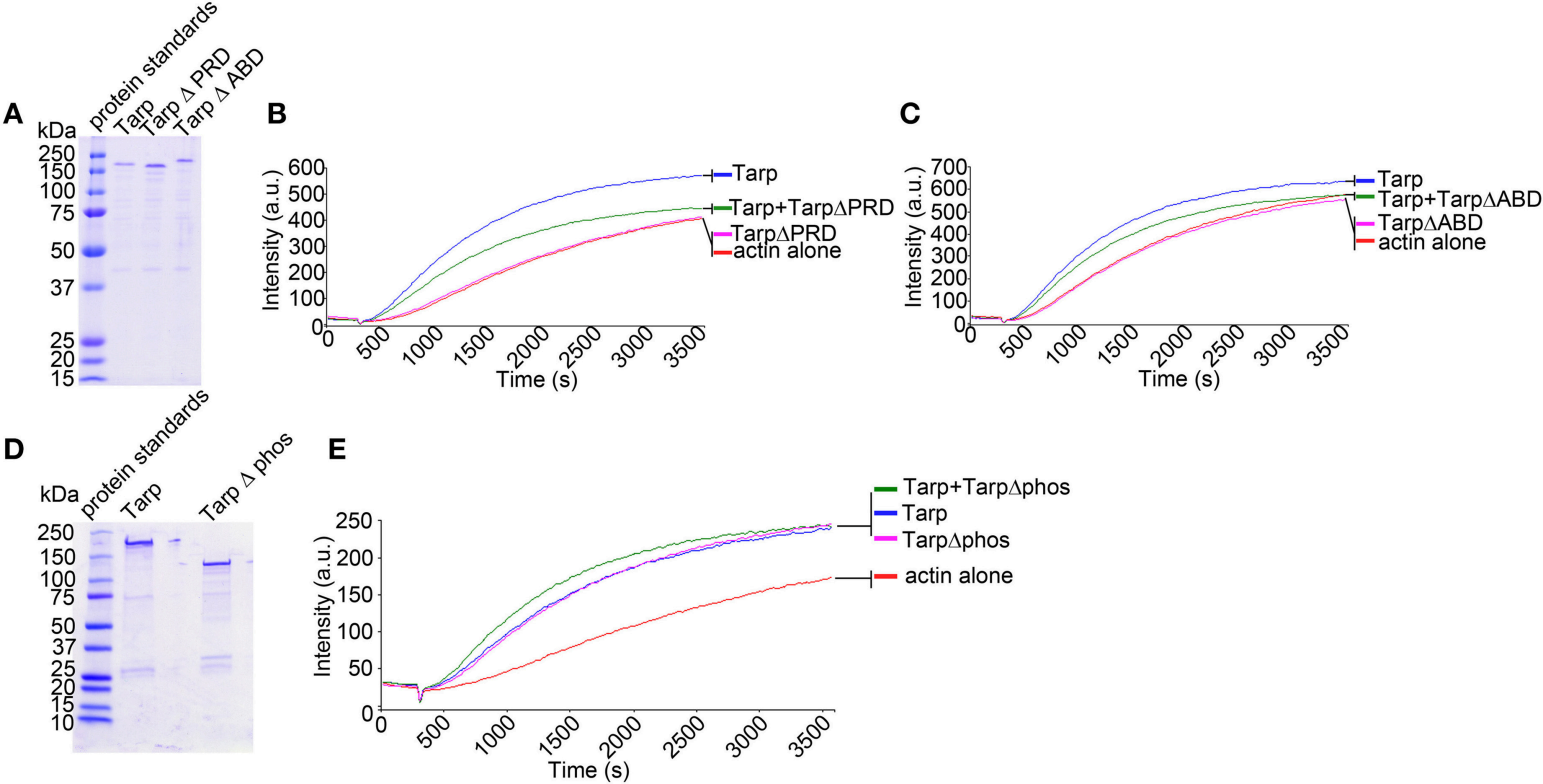
**Tarp mutants inhibit the ability of wild type Tarp to nucleate actin *in vitro*. (A)** Purified Tarp and Tarp mutants harboring deletions in the proline rich oligomerization domain (Δ PRD) and the actin binding domain (Δ ABD). Proteins were resolved by SDS-PAGE and visualized by Coomassie blue staining. **(B)** The Tarp ΔPRD deletion mutant inhibits wild type Tarp mediated actin nucleation in pyrene actin nucleation assays as observed by a decrease in the slope of the pyrene assay. Equal concentrations of proteins described in **(A)** were incubated with 1 μM monomeric pyrene-labeled actin. An increase in actin polymerization after the addition of polymerization buffer at 300 s was measured as arbitrary fluorescence intensity [Intensity (a.u.)] over time [Time(s)]. Pyrene actin alone served as a negative control. **(C)** The Tarp ΔABD deletion mutant also interferes with wild type Tarp mediated actin nucleation in pyrene actin nucleation assays. The experiment was designed as described in **(B)**. using Tarp ΔABD. **(D)** Purified Tarp (as in **A**) and a Tarp mutant harboring a deletion in the tyrosine rich phosphorylation domain (Δphos). Proteins were resolved by SDS-PAGE and visualized by Coomassie blue staining. **(E)** The TarpΔphos deletion mutant enhances wild type Tarp mediated actin nucleation in a pyrene actin nucleation assay as an increase in the slope in the pyrene actin assay was observed. The pyrene curve generated by Tarp Δphos and wild type Tarp was equivalent to a 2× concentration of wild type Tarp curve (data not shown) (experiment was performed similar to **B**,**C**). The pyrene actin polymerization assays are representative of three repeated experiments.

The Tarp **a**ctin **b**inding **d**omain (ABD, amino acids 748–758) and the **p**roline **r**ich polymerization **d**omain (PRD, amino acids 625–650) have previously been found to be required for Tarp mediated polymerization of pyrene actin *in vitro* (a summary of Tarp protein domains is provided in Figure 1; Jewett et al., 2006). One model for Tarp mediated actin nucleation advocates that Tarp oligomerizes via the proline rich domain. Tarp effectors, which come together following secretion into the host cell cytosol, associate with monomeric actin to form an actin nucleus, affectedly reducing the critical concentration of actin required for actin filament formation (Jewett et al., 2006). Since Tarp is hypothesized to function in a homo-oligomeric complex, we tested whether TarpΔPRD and TarpΔABD were able to disrupt wild type Tarp mediated actin polymerization in a dominant negative fashion. To investigate whether mutant Tarps could inhibit actin polymerization induced by wild type Tarp, purified recombinant wild type Tarp proteins and TarpΔABD or TarpΔPRD were combined at equal molar ratios and analyzed for actin nucleation in pyrene actin polymerization assays (Figure 2). Similar to our previous findings, when TarpΔPRD or TarpΔABD alone was introduced to pyrene actin, an increase in the rate of actin polymerization was not observed compared to actin alone controls (Figures 2B,C). The amount of TarpΔPRD employed in these experiments was lower compared to previous studies to ensure that TarpΔPRD would not appreciably sequester monomeric actin (Jiwani et al., 2013). At higher concentrations, TarpΔPRD sequesters monomeric actin and may disrupt native Tarp function by lowering the concentration of available actin in the reaction. As predicted, when (TarpΔPRD or TarpΔABD) were mixed with an equal amount of wild type Tarp, the rate of actin polymerization was reduced (Figures 2B,C). Although the rate of pyrene actin polymerization was reduced in the presence TarpΔPRD or TarpΔABD, some Tarp mediated actin nucleation was still observed. To confirm that the reduction of actin polymerization was not the consequence of steric hindrance caused by excess Tarp in the reaction, an additional control (TarpΔphos) which is not predicted to interfere with actin polymerization was tested. Tarp which lacks the tyrosine rich phosphorylation domain (TarpΔphos, harbors the deletion in amino acids 125–424) has previously been shown to nucleate actin to equivalent rates as compared to wild type Tarp (Jiwani et al., 2013). To test whether Tarp mediated actin polymerization might be affected by TarpΔphos (Figure 2D), purified Tarp and TarpΔphos were mixed at equal molar ratios and tested in pyrene actin polymerization assays. TarpΔphos combined with wild type Tarp enhanced the rate of actin polymerization due to the two-fold molar increase of functional actin nucleators (Figure 2E). These data support the hypothesis that biochemically wild type Tarp may experience reduced actin polymerization kinetics when associated with or competing with defective TarpΔPRD or TarpΔABD. Conversely, wild type Tarp mediated actin polymerization is not inhibited by TarpΔphos.

### *C. trachomatis* transformants are able to express epitope tagged wild type and mutant Tarp effectors

**Figure 3:**
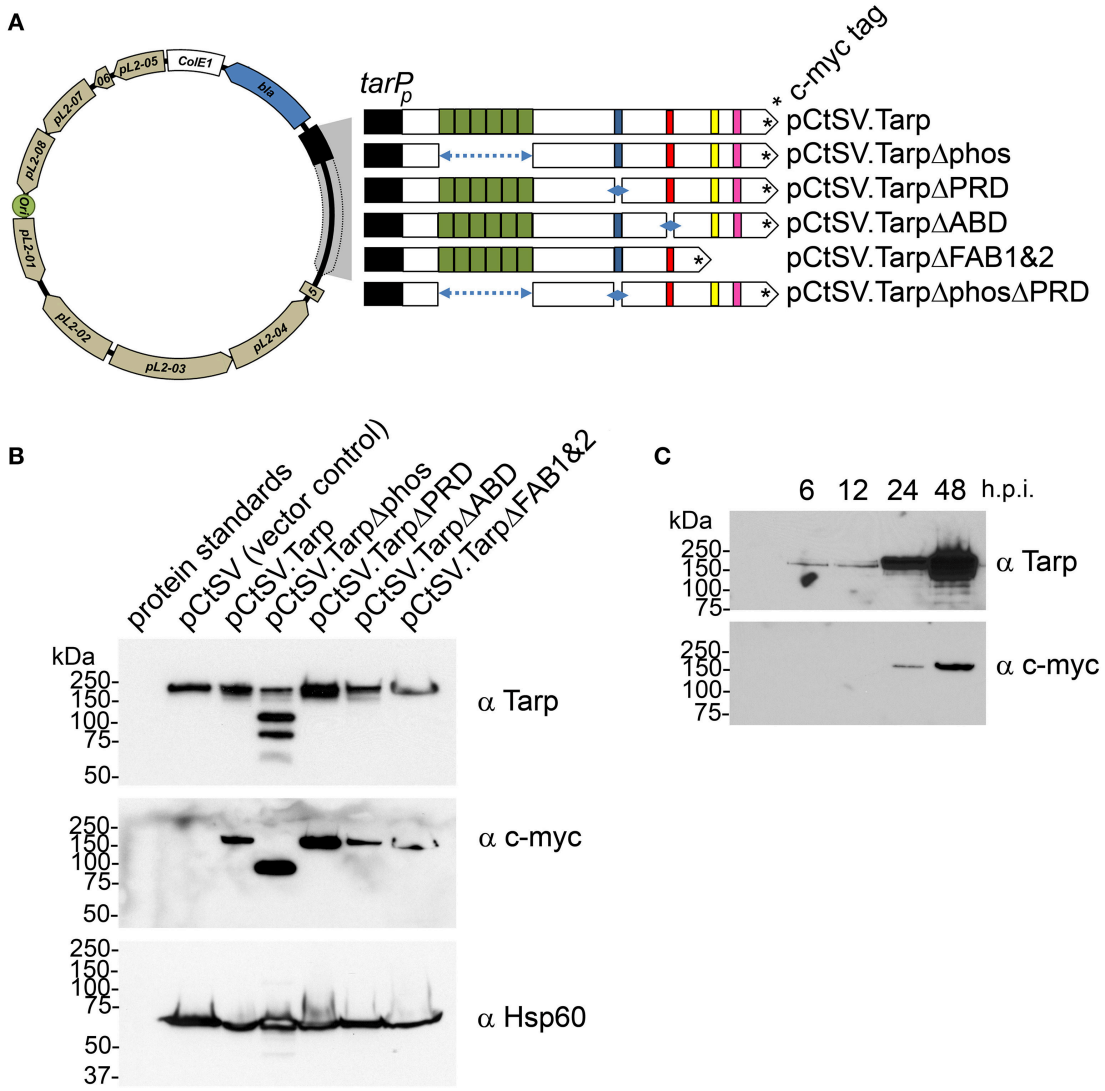
**Schematic diagram of the chlamydial shuttle vector pCtSV.1 and the derivatives engineered to express epitope tagged Tarp mutants. (A)** The *C. trachomatis* shuttle vector pCtSV.1 was adapted to allow for the expression of c-myc tagged Tarp under the control of the *tarP* promoter (*tarP*_*p*_). In frame deletions were generated in the *tarP* gene to remove the phosphorylation domain (pCtSV.TarpΔphos), proline rich domain (pCtSV.TarpΔPRD), g-actin binding domain (pCtSV.TarpΔABD) f-actin binding domains (pCtSV.TarpΔFAB1 and 2), and the double deletion mutant, a phosphorylation domain and proline rich domain mutant (pCtSV.TarpΔphosΔPRD) respectively. **(B)** Transformed *C. trachomatis* express epitope tagged Tarp. Protein lysates were generated from McCoy cells infected with *C. trachomatis* L2 transformed with the shuttle vector pCtSV.1, pCtSVTarp, pCtSV.TarpΔphos, pCtSV.TarpΔPRD, pCtSV.TarpΔABD, and pCtSV.TarpΔFAB1and2 (the shuttle vectors depicted in **A**). Protein samples were resolved by SDS-PAGE and transferred to nitrocellulose membranes for immunoblot analysis with Tarp (α Tarp) and c-Myc (α c-myc) specific antibodies. Molecular mass is in kiloDaltons (kDa). **(C)** *C. trachomatis* (+pCtSV.Tarp) Infected host cells were collected from a 6 well plate at 6, 12, 24, and 48 h post infection and solubilized in protein sample buffer. Protein samples were resolved by SDS-PAGE and transferred to nitrocellulose membranes for immunoblot analysis with Tarp (α Tarp) and c-Myc (α c-myc) specific antibodies.

In order to determine whether mutant Tarp effectors might disrupt endogenous Tarp function *in vivo*, we engineered a chlamydial shuttle vector to express epitope tagged wild type and mutant Tarp effectors (Figure 3A). *C. trachomatis* genes are temporally regulated so as to match their function with the correct window in the chlamydial developmental cycle. To promote coordinated expression of the mutant Tarp alleles with that of the endogenous Tarp gene, mutant Tarp effectors expressed from the chlamydial shuttle vector were engineered under the control of ~200 nucleotides of DNA upstream of the annotated Tarp gene, which we have termed the Tarp promoter (*tarP*p) (Figure 3). Mutant Tarp constructs included deletions of the phosphorylation domain, the proline rich domain, the actin binding domain and a Tarp truncation which resulted in the deletion of the F-actin binding domains 1 and 2 (Figure 3A). *C. trachomatis* L2 transformed with each shuttle vector were expanded under antibiotic selection for several passages, density gradient purified and tested for the presence of the c-myc epitope tag by western blot analysis. All of the transformants produced c-myc tagged proteins of the expected size (Figure 3B). Interestingly, endogenous Tarp in *C. trachomatis* expressing TarpΔphos appears to be lower compared to the relative abundance of Tarp in the other transformants. This may hold true for the other transformants (ABD, PRD, and FAB1 and 2) but cannot be visualized in the western analysis since mutant and endogenous Tarp migrate to the same position on the protein gels.

### *C. trachomatis* transformants expressing mutant Tarp effectors are inhibited in bacterial invasion of host cells

**Figure 4:**
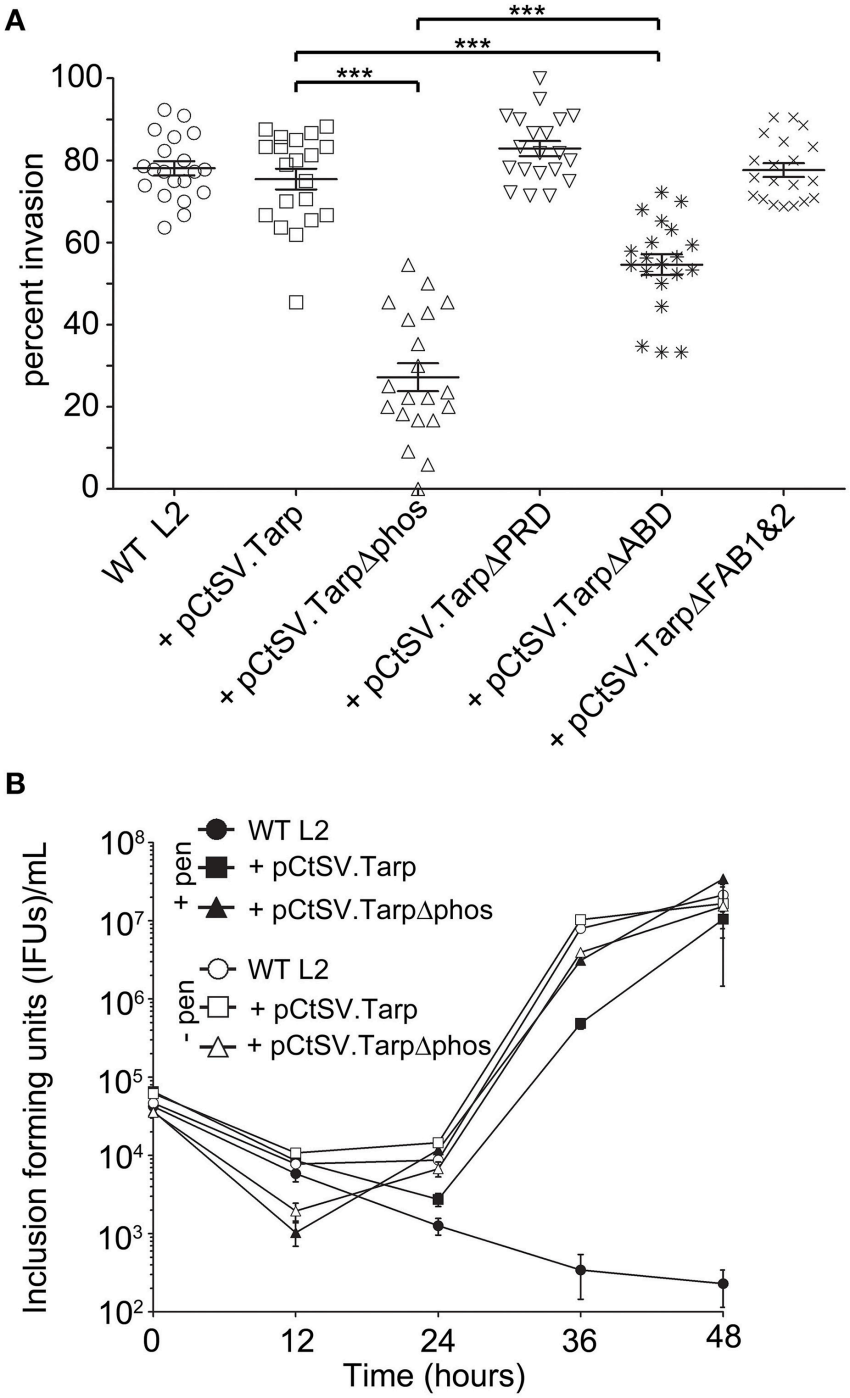
***C. trachomatis* transformants harboring epitope tagged mutant Tarps are deficient in chlamydial entry. (A)** Wild type *C. trachomatis* (L2; circles) or L2 transformants harboring plasmid pCtSV.Tarp (+ pCtSV.Tarp; squares), pCtSV.TarpΔphos (+ pCtSV.TarpΔphos; triangles), pCtSV.TarpΔPRD (+ pCtSV.TarpΔPRD; inverted triangles), pCtSV.TarpΔABD (+ pCtSV.TarpΔABD; asterisks), or pCtSV.TarpΔFAB1and2 (+ pCtSV.TarpΔFAB1and2; “x”), were examined for chlamydial invasion of HeLa 229 cells. Intrinsically fluorescent cell tracker (CMPTX) labeled EBs were used in invasion assays. After allowing 1 h for invasion, extracellular EBs were counterstained by indirect immunofluorescence with a monoclonal antibody to *C. trachomatis* L2 MOMP and a goat anti mouse antibody conjugated to Alexa 488. The data are represented as the percentage of intracellular EBs relative to the total number of extracellular and intracellular EBs per field of view. The data represented from two biological replicates are shown. Each data point represents a single field of view at 1000 × magnification. Data sets were compared with one way ANOVA and Tukey's multiple comparison test of the mean. ^***^represents a *p* < 0.001. **(B)** Development of wild type *C. trachomatis* L2 (circles) and transformants harboring plasmid pCtSV.Tarp (+ pCtSV.Tarp; squares), pCtSV.TarpΔphos (+ pCtSV.TarpΔphos; triangles), after normalizing the initial multiplicity of infection for each clone (IFU normalization was confirmed by determining the number of inclusions formed at time zero). Infected cells with antibiotic selection (filled symbols) and infected cells without antibiotic selection (open symbols) were collected at *t* = 0, 12, 24, 36, and 48 h post infection and mechanically lysed to release infectious EBs. Inclusion forming units (IFUs) were determined for each transformant by serial dilution of released EBs harvested at each time point and reinfection of HeLa cells grown on coverslips to determine the number of inclusion forming units per mL of harvested material.

We hypothesized that production of a dominant negative Tarp complex *in vivo* would have a significant effect on the ability of elementary bodies to invade host cells. To test the invasion potential of the five different *C. trachomatis* clones expressing mutant Tarp effectors from the *C. trachomatis* shuttle vector, we performed invasion assays to quantitate the number of elementary bodies which entered a host cell in a 1 h time period (Figure 4A). As predicted, *C. trachomatis* expressing Tarp lacking the actin binding domain (TarpΔABD) demonstrated a significant reduction in host cell invasion compared to wild type *C. trachomatis* L2 and *C. trachomatis* harboring pCtSV.Tarp. Surprisingly and in contrast to the *in vitro* actin polymerization studies, *C. trachomatis* expressing TarpΔphos displayed a significant reduction in invasion relative to all of the clones analyzed, including *C. trachomatis* expressing TarpΔABD (Figure 4A). No altered invasion phenotype was observed for *C. trachomatis* expressing TarpΔPRD or TarpΔFAB1 and 2. In order to examine if the presence of a dominant-negative Tarp might disrupt chlamydial development, growth curves were performed for *C. trachomatis* expressing TarpΔphos and wild type controls (Figure 4B). No significant changes to chlamydial growth was observed between wild type and *C. trachomatis* expressing TarpΔphos as measured by the number of EBs harvested from infected cells at 0, 12, 24, 36, and 48 h post infection (Figure 4B and data not shown).

### *C. trachomatis* expressing TarpΔphos demonstrate reduced phosphorylation of endogenous tarp

**Figure 5:**
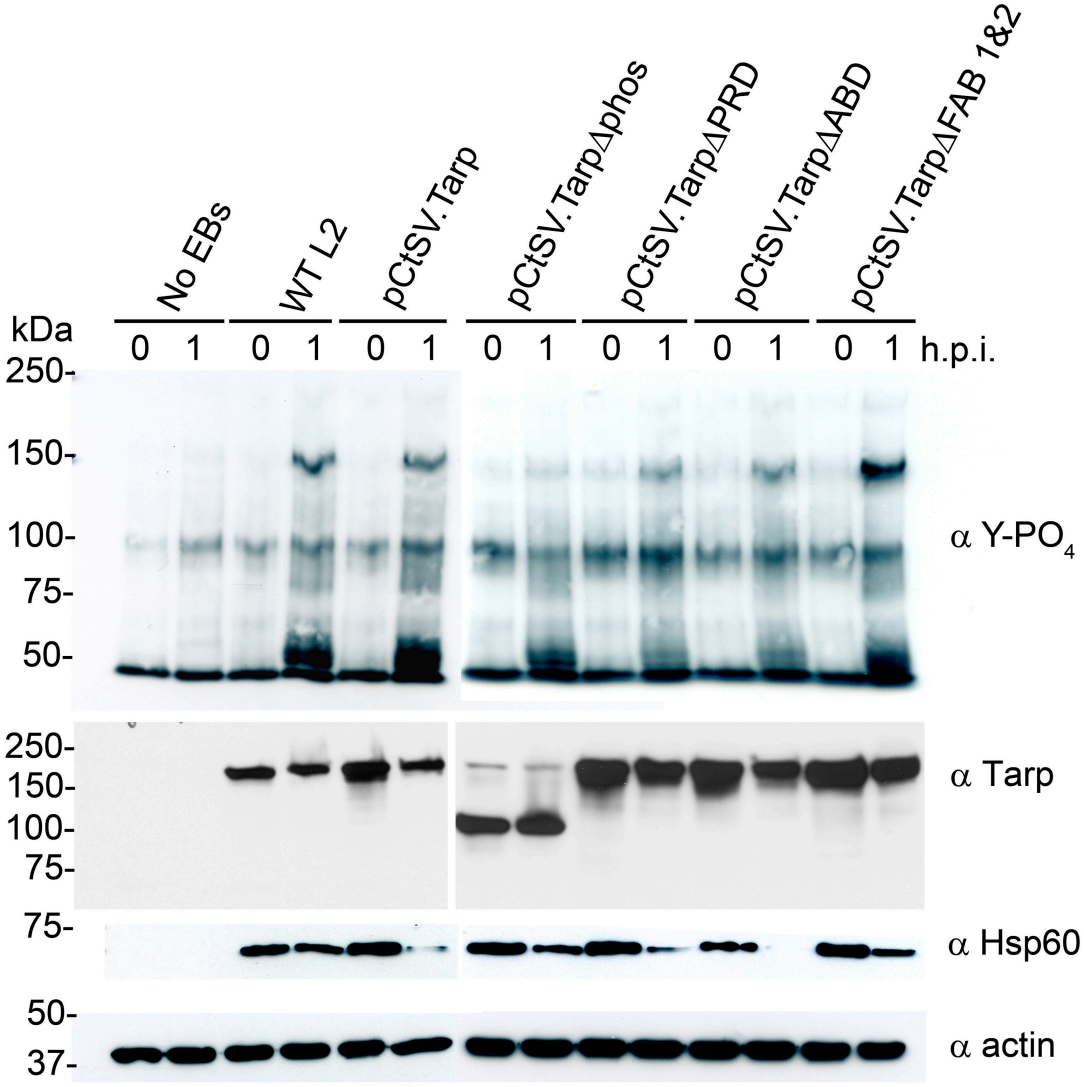
***C. trachomatis* expressing TarpΔphos exhibit reduced levels of tyrosine phosphorylation during entry**. Wild type *C. trachomatis* (WT L2) or L2 transformants harboring plasmids pCtSV.Tarp pCtSV.TarpΔphos, pCtSV.TarpΔPRD, pCtSV.TarpΔABD or pCtSV.TarpΔ FAB1and2 were used to infect McCoy host cells for 1 h. Mock treated host cells (No EBs) served as a negative control. Infected host cells were collected from a 6 well plate at 0 and 1 h post infection and solubilized in protein sample buffer. Protein samples were resolved by SDS-PAGE and transferred to nitrocellulose membranes for immunoblot analysis with phosphotyrosine (α Y-PO_4_), chlamydial heat shock protein 60 (α Hsp60), Tarp (α Tarp) and actin (α actin) specific antibodies. The infection and subsequent phosphorylation western blot assay presented is representative of three repeated experiments.

We hypothesized that *C. trachomatis* expressing TarpΔphos might show a reduction in endogenous Tarp phosphorylation following attachment and entry into host cells as TarpΔphos is likely to compete with endogenous Tarp for translocation into the host cell. TarpΔphos is missing the tyrosine residues known to be phosphorylated by Src family kinases (SFKs) therefore TarpΔphos is not expected to be phosphorylated by host cell tyrosine kinases after translocation into the host cell. In order to test this, wild type *C. trachomatis* or *C. trachomatis* harboring the plasmids presented in Figure 3 were added to host cells and protein lysates were generated at 0 and 1 h post infection. Tarp has previously been identified as the predominant phosphotyrosine modified protein of ~150 kDa which is observed in the wild type *C. trachomatis* infected cells. Interestingly, phosphorylated Tarp was reduced in those bacteria expressing TarpΔphos, but not in the other transformants (Figure 5).

### *C. trachomatis* is able to translocate TarpΔphos into the host cells

**Figure 6:**
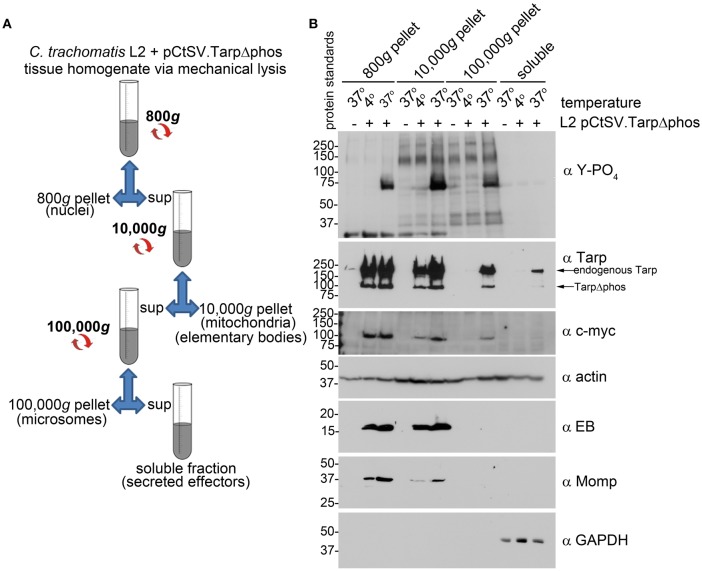
***C. trachomatis* transformants demonstrate secretion of both wild type Tarp and epitope tagged mutant TarpΔphos. (A)** The schematic of the differential centrifugation steps and the supernatants (sup) and pellets recovered from centrifugation at 800, 10,000, and 100,000 × g. Secreted effector proteins such as Tarp are expected to be detectable in the bacteria-free 100,000 × g pellet (microsomes) and soluble fractions. **(B)** Subcellular fractionation of *C. trachomatis* infected cells by differential centrifugation out to 100,000 × g yields a soluble Tarp fraction that is distinct from intact elementary bodies. Total lysates derived from McCoy host cells infected with *C. trachomatis* serovar L2 transformed with the shuttle vector pCtSV.TarpΔphos (L2 pCtSV.TarpΔphos) underwent subcellular fractionation by centrifugation. Fractions were resolved by SDS-PAGE and transferred to nitrocellulose for immunoblot analysis with antibodies specific for phosphotyrosine (α Y-PO_4_), Tarp (α Tarp), c-Myc epitope (α c-myc), elementary bodies (α EB), *C. trachomatis* major outer membrane protein (α Momp), Glyceraldehyde 3-phosphate dehydrogenase a soluble protein marker (α GAPDH) and actin a protein expected to be present in all fractions (α actin). The infection and subsequent cell fractionation data presented is representative of three repeated experiments.

*C. trachomatis* expressing both endogenous Tarp and TarpΔphos demonstrated a reduction in host cell invasion and Tarp phosphorylation. These results raised the possibility that type three secretion is altered and/or inhibited in *C. trachomatis* expressing TarpΔphos. To determine whether *C. trachomatis* expressing TarpΔphos was capable of type three secretion we examined whether endogenous Tarp and plasmid encoded epitope tagged TarpΔphos could be recovered from the soluble fraction following subcellular fractionation of host cells (Figure 6A). The fractionation profile of wild type *C. trachomatis* infected cells is presented as Supplemental Figure 1 for reference. Endogenous Tarp and epitope tagged TarpΔphos can be distinguished by their unique molecular weights and the presence or absence of the c-myc epitope tag (Figure 3). Protein supernatant samples sequentially obtained from 800, 10,000, and 100,000 × g centrifugal spins indicated that the endogenous Tarp and TarpΔphos effectors co-fractionated and were detectable in fractions that were distinct from intact EBs, which pellet at 10,000 × g (Figure 6B). The endogenous Tarp and TarpΔphos proteins were observed in the 100,000 × g pellet and the 100,000 × g soluble fraction, the latter represents the host cell cytosolic fraction as defined by the presence of the soluble eukaryotic protein GAPDH (Figure 6B). We hypothesize that most of the secreted tarp is restricted to the 100,000 × g pellet due to interactions with actin filaments (short actin filaments pellet at 100,000 × g). The portion of Tarp found in the 100,000 × g supernatant (soluble fraction) may represent those proteins which have not yet associated with host cell molecules of the cytoskeleton. Non-secreted chlamydial antigens identified by the anti-EB and anti-MOMP antibodies were not detected in fractions beyond the 10,000 × g pellet, indicating that these later protein fractions did not contain lysed EBs. It has been shown previously that type three secretion by *C. trachomatis* is temperature dependent and can be inhibited at low temperatures (Jamison and Hackstadt, 2008). Consistent with this finding, endogenous and mutant Tarp were not detected in the host cell cytosolic fraction of host cells maintained at 4°C during the course of the *C. trachomatis* infection. Together these data indicate that *C. trachomatis* expressing TarpΔphos is capable of type three secretion mediated delivery of endogenous and mutant Tarp into the host cell cytosol. Although the data presented in Figure 6B do not provide quantitative measures of WT and TarpΔphos secretion, the experiment indicates that both endogenous and TarpΔphos are actively secreted into the host cell.

### Dissociation of TarpΔphos from the native Tarp effector is able to restore *C. trachomatis* invasion of host cells

**Figure 7:**
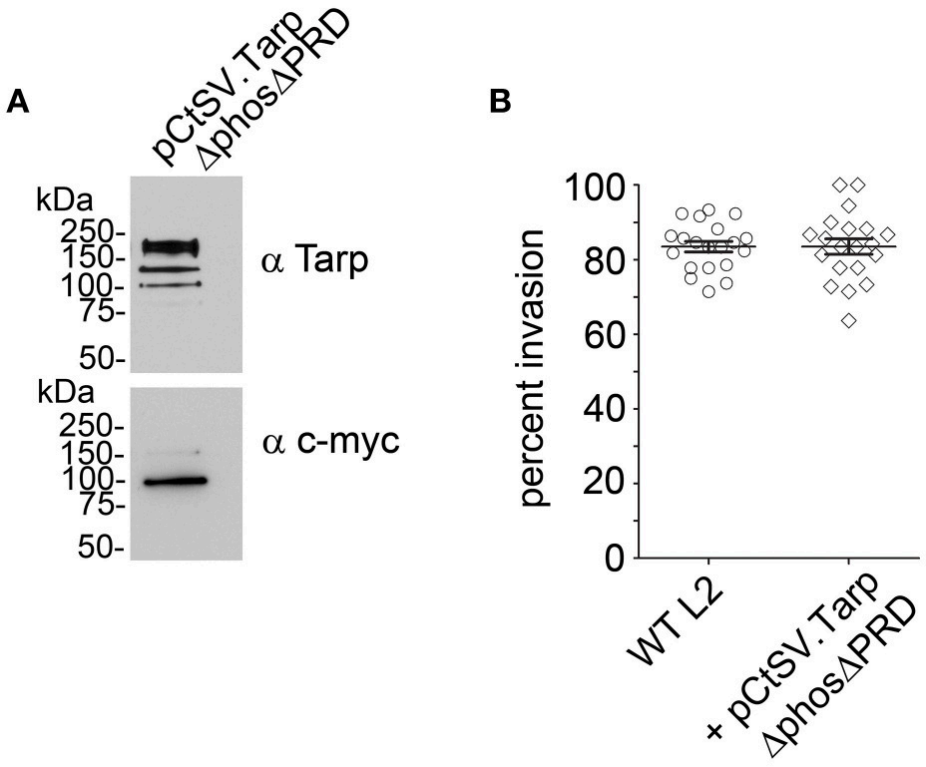
**Introduction of a second protein domain deletion within TarpΔphos disrupts the dominant negative phenotype**. Since the proline rich domain (PRD) is implicated in Tarp oligomerization, a PRD domain deletion was introduced into the pCtSV.TarpΔphos shuttle vector creating pCtSV.TarpΔphosΔPRD which is engineered to express an epitope tagged double mutant TarpΔphosΔPRD. **(A)** Protein samples were resolved by SDS-PAGE and transferred to nitrocellulose membranes for immunoblot analysis with Tarp (α Tarp) and c-Myc (α c-myc) specific antibodies. Molecular mass is in kiloDaltons (kDa). **(B)** As described previously, *C. trachomatis* transformants were examined for bacterial invasion of host cells. EBs were examined for chlamydial invasion of HeLa 229 cells. Intrinsically fluorescent cell tracker (CMPTX) labeled EBs were used in invasion assays. After allowing 1 h for invasion, extracellular EBs were counterstained by indirect immunofluorescence with a monoclonal antibody to *C. trachomatis* L2 MOMP and a goat anti mouse antibody conjugated to Alexa 488. The data are represented as the percentage of intracellular EBs relative to the total number of extracellular and intracellular EBs per field of view. The data from two biological replicates are shown. Each data point represents a single field of view at 1000 × magnification.

Previous studies with purified recombinant Tarp effectors revealed that Tarp multimerization is mediated by a region of ~25 amino acids rich with prolines subsequently called the proline rich domain (PRD) (Jewett et al., 2006). This model suggests that plasmid encoded TarpΔphos and endogenous Tarp may associate following translocation into the cell perhaps resulting in the observed dominant-negative invasion phenotype. To further test this hypothesis, we sought to disrupt the ability of TarpΔphos to form heteromeric complexes with wild type Tarp through genetic deletion of the proline rich domain from the pCtSV TarpΔphos construct. According to our model, a TarpΔphosΔPRD double mutant lacking both the phosphorylation domain and the proline rich oligomerization domain would be unable to associate with endogenous Tarp, rescuing the dominate negative phenotype caused by the TarpΔphos protein. As predicted, C. trachomatis transformants harboring pCtSVTarpΔphosΔPRD demonstrated wild type levels of chlamydial entry after 1 h (80% internalized EBs) and displayed no defect in chlamydial growth (Figure 7 and data not shown).

## Discussion

Obligate intracellular pathogens such as *C. trachomatis* harbor unique tools to hijack host cell processes, promoting bacterial replication and immune evasion. The Tarp effector is a candidate virulence factor that is hypothesized to trigger host cell entry and other host signaling events to promote pathogen invasion. By leveraging our understanding of the biochemical features of the Tarp polypeptide, we now demonstrate that mutant Tarp effectors can be engineered to disrupt pathogen entry of host cells in a dominant-negative manner. The interplay between the host and the pathogen is intricate and involves mechanisms of pathogen-targeted disruption of host cellular processes, which are only partially understood (Ribet and Cossart, 2015). To some degree, *C. trachomatis* execute cellular override mechanisms by delivering bacterial effector proteins into the host cell cytosol via a type three secretion apparatus (Ferrell and Fields, 2016). Consequentially, a number of studies have focused on how Tarp is able to associate with host cell actin, tyrosine kinases, and SH2 domain containing proteins (Jewett et al., 2006, 2008; Lane et al., 2008; Mehlitz et al., 2008, 2010; Lutter et al., 2010; Jiwani et al., 2012, 2013). Tarp is a large protein consisting of 1005 amino acids and is, biochemically speaking, one of the most well characterized *C. trachomatis* effectors (Mueller et al., 2014). Host cytoskeletal rearrangements are required for bacterial invasion of host cells and it is believed that *C. trachomatis* actively directs this process, mediated in part by Tarp translocation into the host cell. Recombinant Tarp by itself is a potent nucleator of actin and dramatically increases the rate of actin polymerization compared to actin alone controls in *in vitro* pyrene actin polymerization assays (Jewett et al., 2006). Domain deletion analysis has revealed the minimum protein sequence required for actin binding and actin nucleation (Jewett et al., 2006). These studies demonstrated that Tarp mediated actin nucleation is predominantly driven by Tarp oligomerization, which could make this biochemical function amenable to interference or disruption if the Tarp complex failed to assemble appropriately *in vivo* (Jewett et al., 2006). With the advent of a chlamydia transformation system (Wang et al., 2011), it was now possible to genetically manipulate *C. trachomatis* to express plasmid-encoded mutant Tarp effectors engineered to disrupt endogenous Tarp-mediated actin nucleation. As hypothesized, *C. trachomatis* producing a mutant Tarp effector, which lacked the actin binding domain required for actin nucleation, resulted in disruption of chlamydial entry into host cells. These findings were consistent with the ability of purified TarpΔABD protein to reduce the actin nucleation activity of purified wild type Tarp protein *in vitro.* Together these data suggest that *C. trachomatis* producing both endogenous Tarp and TarpΔABD protein have a diminished ability to nucleate actin resulting in reduced host cell invasion. Conversely, *C. trachomatis* harboring mutant Tarp effectors lacking the proline rich domain required for Tarp oligomerization did not disrupt bacterial entry. This may not be surprising as TarpΔPRD is predicted to lack the ability to associate with endogenous Tarp and therefore is not likely to disrupt the homomeric complex critical for Tarp function. Purified TarpΔPRD alone can sequester monomeric actin in a concentration dependent manner *in vitro* (Figure 2B; Jiwani et al., 2013). The lower rate of actin polymerization observed for the combination of purified TarpΔPRD and purified wild type Tarp *in vitro* may be the result of reduced monomeric actin available in the pyrene assay due to sequestration of monomeric actin by TarpΔPRD protein. Although it remains a possibility that TarpΔPRD produced by the *C. trachomatis* pCtSVTarpΔPRD clone is able to sequester monomeric actin *in vivo*, this ability does not result in a measurable change in host cell invasion.

Interestingly, the greatest inhibition of EB entry of host cells was observed for *C. trachomatis* transformants which expressed TarpΔphos. Based on our *in vitro* pyrene actin polymerization assays it is unlikely that TarpΔphos is disrupting the direct actin nucleation activity of endogenous Tarp. Tarp phosphorylation has been implicated in host cell signaling via SH2 domain containing host cell proteins that promote the activation of other host cell actin nucleators such as the Arp2/3 complex (Lane et al., 2008; Mehlitz et al., 2010). Therefore, a heterocomplex between TarpΔphos and endogenous wild type Tarp may indirectly disrupt actin nucleation as a result of altered host cell signaling. Previous experiments have indicated that phosphorylated Tarp is unable to active the Arp2/3 complex directly and that any host cell signaling cascades that may be initiated by phosphorylated Tarp also requires activation of nucleation promoting factors (NPFs) such as WASP/N-WASP (Jiwani et al., 2012). Despite the compelling biochemical evidence for the indirect contribution of Tarp phosphorylation to actin polymerization and host cell entry, the role for Tarp phosphorylation in *C. trachomatis* invasion remains unclear. In contrast to the invasion phenotype conferred by *C. trachomatis* expressing TarpΔphos, it has been shown that inhibition of Tarp phosphorylation via tyrosine kinase inhibitors such as PP2 does not significantly inhibit *C. trachomatis* entry into host cells (Jewett et al., 2008). A key difference between these two studies is the chemical inhibition of phosphorylation of full length endogenous Tarp versus phenotypic analysis of *C. trachomatis* transformants harboring Tarp molecules which are missing the phosphorylation domain. It is therefore possible that the tyrosine rich repeat region is not only important for Tarp phosphorylation, but has an additional undescribed role in EB entry. The data presented herein suggest that the presence of TarpΔphos has altered endogenous Tarp function leading to reduced *C. trachomatis* invasion or that TarpΔphos has disrupted EB entry independently. We found no evidence for significantly altered levels of Tarp secretion as our cell fractionation experiments revealed that both endogenous Tarp and TarpΔphos were isolated from the soluble fractions containing the host cell cytosol alone. Although it remains to be seen if subtle quantitative differences in Tarp concentration or in secretion relative to wild type may have a profound effect on bacterial invasion. Previous reports have demonstrated that Tarp secretion is mediated by the chlamydial chaperone Slc1 (Brinkworth et al., 2011). Slc1 and Tarp associate via the N-terminal domain of Tarp mapped to amino acids 1–200. Interestingly, TarpΔphos lacks amino acids 125–424 and was still capable of translocation into the host cell, therefor TarpΔphos further delineates the region required for T3SS-mediated secretion of Tarp.

Tarp proteins form oligomeric complexes *in vitro* which are mediated by the proline rich domain. In support of this model, deletion of the proline rich domain from the TarpΔphos protein restored EB invasion to wild type levels. Tarp phosphorylation is implicated in host cell signaling; although these signaling mechanisms have not previously been experimentally defined to be directly associated with entry, our data now suggest that the Tarp tyrosine rich sequence that comprises the phosphorylation domain itself is important for entry.

Analysis of Tarp orthologs from different chlamydial species and serovars has revealed conserved biochemical features such as the ability to bind and nucleate actin albeit by engaging slightly different mechanisms (Jewett et al., 2010). Conversely, many unique characteristics, such as *C. trachomatis* L2 Tarp phosphorylation or *C. caviae* strain GPIC Tarp's ability to bind to focal adhesion kinase (FAK) reveal that Tarp orthologs may have evolved to serve specific functions for unique human versus animal hosts (Clifton et al., 2005; Thwaites et al., 2014). The ability to introduce Tarp deletion mutants into *C. trachomatis* is the first step toward defining which protein domains may play a dominant role *in vivo*. This work may be expanded in the future to include analysis of other Tarp orthologs as well as the generation of a Tarp null or a conditional knockout.

A mechanistic understanding of chlamydial entry of host cells will likely lead to novel interventions that prevent *C. trachomatis* infections. Many questions still remain, but this work represents the first instance of the *in vivo* analysis of the Tarp effector and its functional domains within EBs and the utilization of dominant negative mutant alleles to disrupt chlamydial invasion of host cells.

## Author contributions

CP: data acquisition, data analysis, data interpretation, revising of the manuscript; RL: data acquisition, data analysis, data interpretation, revising of the manuscript; BN: data acquisition, data analysis, data interpretation; LR: data acquisition, data analysis, data interpretation; TJ: data acquisition, data analysis, data interpretation, writing of the manuscript, revising of the manuscript, principle investigator.

## Funding

This work was supported by the National Institute of Allergy and Infectious Diseases of the National Institutes of Health under award number R21AI117013. The content is solely the responsibility of the authors and does not necessarily represent the official views of the National Institutes of Health.

### Conflict of interest statement

The authors declare that the research was conducted in the absence of any commercial or financial relationships that could be construed as a potential conflict of interest.

## Supplementary material

Supplementary Figure1 **Subcellular fractionation of wild type *C. trachomatis* infected cells yields a phosphorylated Tarp fraction distinct from intact elementary bodies**. HeLa 229 cells infected with wild type *C. trachomatis* serovar L2 (L2) underwent subcellular fractionation by centrifugation of lysed cells (total lysate). Lysate pellets were sequentially obtained from 800, 10,000, and 100,000 × g spins leaving a 100,000 × g supernatant (soluble fraction) Fractions were resolved by SDS-PAGE and transferred to nitrocellulose for immunoblot analysis with antibodies specific for phosphorylated tyrosines (α Y-PO4), Tarp (α Tarp), elementary bodies (α EBs) Glyceraldehyde 3-phosphate dehydrogenase (α GAPDH), actin (α actin), lysosome associated membrane protein 1 (α Lamp1) and Phosphoinositide 3-kinase (α PI3K).
