# Supplementary figures and images for: Targeted Disruption of Chlamydia trachomatis Invasion by in Trans Expression of Dominant Negative Tarp Effectors
Source: Front Cell Infect Microbiol. 2016 Aug 23;6:84. doi: 10.3389/fcimb.2016.00084 (PMC4993794; doi:10.3389/fcimb.2016.00084)

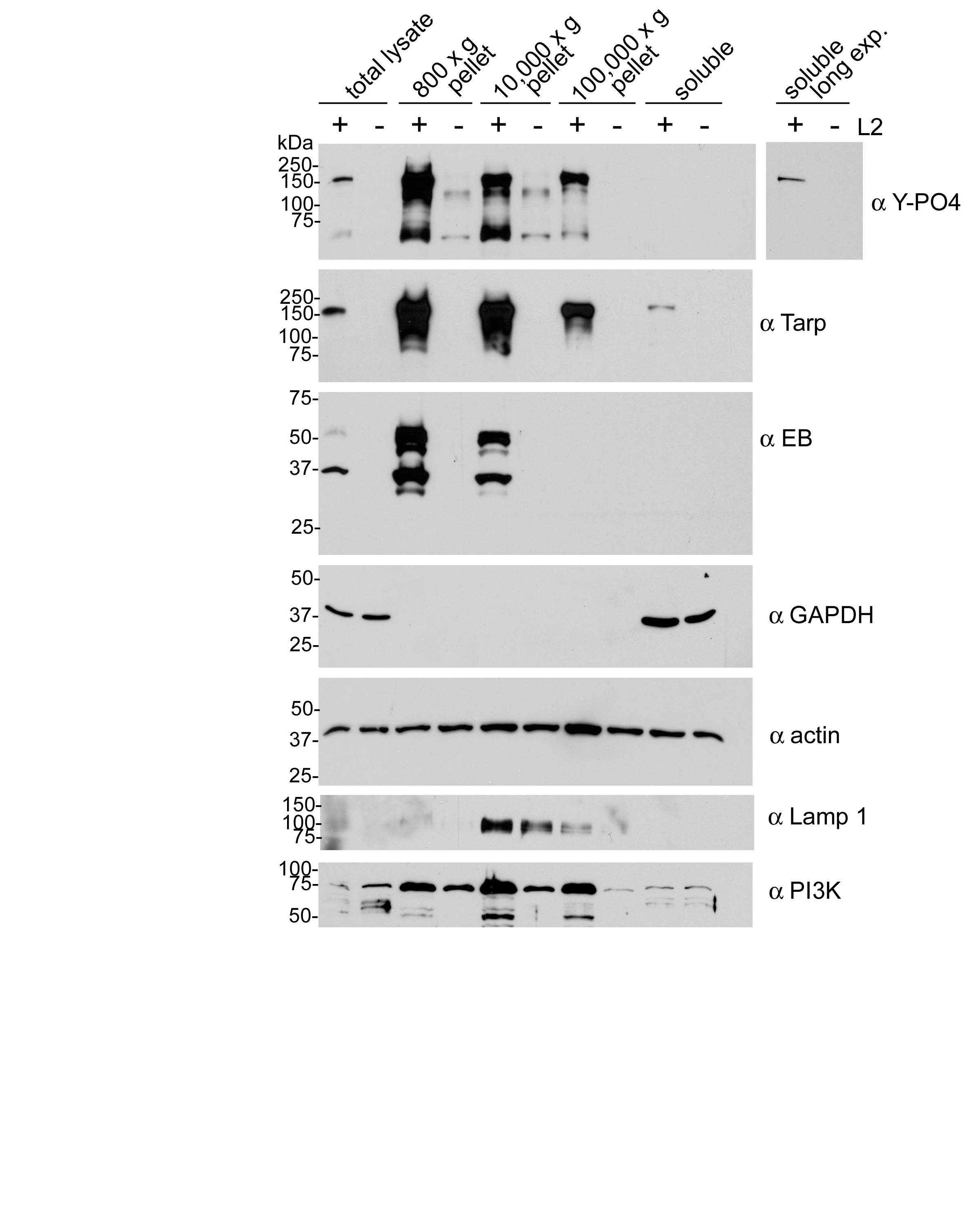

Supplement: Supplementary Figure1 — Subcellular fractionation of wild type C. trachomatis infected cells yields a phosphorylated Tarp fraction distinct from intact elementary bodies. HeLa 229 cells infected with wild type C. trachomatis serovar L2 (L2) underwent subcellular fractionation by centrifugation of lysed cells (total lysate). Lysate pellets were sequentially obtained from 800, 10,000, and 100,000 × g spins leaving a 100,000 × g supernatant (soluble fraction) Fractions were resolved by SDS-PAGE and transferred to nitrocellulose for immunoblot analysis with antibodies specific for phosphorylated tyrosines (α Y-PO4), Tarp (α Tarp), elementary bodies (α EBs) Glyceraldehyde 3-phosphate dehydrogenase (α GAPDH), actin (α actin), lysosome associated membrane protein 1 (α Lamp1) and Phosphoinositide 3-kinase (α PI3K). [file Image1.JPEG]
